# Predictive Value of Platelet-Based Indexes for Mortality in Sepsis

**DOI:** 10.3390/biomedicines14010211

**Published:** 2026-01-19

**Authors:** Alice Nicoleta Drăgoescu, Adina Turcu-Stiolica, Marian Valentin Zorilă, Bogdan Silviu Ungureanu, Petru Octavian Drăgoescu, Andreea Doriana Stănculescu

**Affiliations:** 1 Department of Anesthesiology and Intensive Care, Faculty of Medicine, University of Medicine and Pharmacy of Craiova, 200349 Craiova, Romania; alice.dragoescu@umfcv.ro; 2 Department of Management and Pharmaceutical Marketing, Faculty of Pharmacy, University of Medicine and Pharmacy of Craiova, 200349 Craiova, Romania; adina.turcu@umfcv.ro; 3 Department of Legal Medicine, Faculty of Medicine, University of Medicine and Pharmacy of Craiova, 200349 Craiova, Romania; 4 Department of Gastroenterology and Hepatology, University of Medicine and Pharmacy of Craiova, 200349 Craiova, Romania; bogdan.ungureanu@umfcv.ro; 5 Department of Urology, Faculty of Medicine, University of Medicine and Pharmacy of Craiova, 200349 Craiova, Romania; octavian.dragoescu@umfcv.ro

**Keywords:** sepsis, platelet indices, prognostic biomarkers, mortality prediction

## Abstract

**Background:** Even though there have been improvements in antimicrobial and supportive therapies, sepsis and septic shock are still major causes of death in intensive care units. Early prognostic stratification is very important for helping doctors make decisions. Platelet-derived indices may provide useful, low-cost indicators that signify both inflammatory activation and coagulation irregularities. This study looked at how well different platelet-based ratios could predict death in the hospital from sepsis. **Materials and Methods:** We performed a prospective observational study spanning one year in a tertiary ICU, enrolling 114 adult patients diagnosed with sepsis or septic shock. Upon admission, four platelet-related biomarkers were measured: the C-reactive protein-to-platelet ratio (CPR), the platelet-to-lymphocyte ratio (PLR), the platelet-to-white blood cell ratio (PWR), and the platelet-to-creatinine ratio (PCR). Logistic regression models and receiver operating characteristic (ROC) analyses were employed to assess predictive accuracy. **Results:** Compared to survivors, non-survivors (n = 39) had much higher CRP levels and CPR values, alongside lower platelet and lymphocyte counts. The CPR index showed the best ability in differentiating between non-survivors and survivors (AUC 0.757), with a best cutoff of 0.886. In simplified multivariate models, CPR was still an independent predictor of death in the hospital (OR 1.98; 95% CI 1.22–3.21), whereas PLR and PWR were not. PCR showed a non-significant trend toward lower values in not survivors. **Conclusions:** CPR is a strong and clinically viable predictor of early mortality in sepsis, outperforming other platelet-based indices. Derived from routine laboratory parameters, CPR serves as a valuable adjunct for initial risk stratification in the ICU. To further confirm its prognostic role and incorporation into current scoring systems, large-scale multicenter studies with longitudinal measurements are warranted to validate its prognostic utility and integration into existing scoring systems.

## 1. Introduction

According to the Sepsis-3 criteria, sepsis is life-threatening organ dysfunction caused by an uncontrolled response of the host to infection [1]. Even with improvements in antimicrobial therapy and critical care management, sepsis is still a major global health problem and one of the main causes of death and illness in intensive care units (ICUs) [2]. It is very important to find patients who are at risk of getting worse quickly as soon as possible. This is because timely intervention can greatly lower the chances of septic shock and improve outcomes.

Current clinical tools like the Sequential Organ Failure Assessment (SOFA) score and its simpler version, qSOFA, help figure out how bad a disease is, but they are not very good at predicting early death or helping doctors make quick decisions [3]. As a result, inexpensive and easily accessible haematologic parameters have garnered interest for their potential prognostic significance in sepsis. The neutrophil-to-lymphocyte ratio (NLR) [4,5], platelet count, red cell distribution width (RDW), and mean platelet volume (MPV) have demonstrated correlations with the severity and outcomes of sepsis [6]. These markers are especially important in the ICU, where there is an urgent need for quick, cheap tools that show both inflammation and organ failure.

Emerging evidence suggests that specific blood cells ratios may serve as integrated markers of systemic stress. For example, elevated NLR values are associated with increased mortality, multiple organ failure, and prolonged ICU length of stay in septic patients [7]. The platelet-to-lymphocyte ratio (PLR) may reflect platelet activation and vascular inflammation, both of which lead to microcirculatory dysfunction [8]. In contrast, the monocyte-to-lymphocyte ratio has been linked to prognosis in both infectious and non-infectious critical illness [9]. In addition to cellular indices, biochemical ratios that include CRP, albumin, creatinine, or urea have been suggested as indicators that integrate data on inflammation, nutrition, and organ damage [10,11].

Platelets play a pivotal role in sepsis pathophysiology, transcending their traditional haemostatic roles [12]. Platelets get activated by inflammatory mediators, endotoxins, and interactions between immune cells. This leads to the formation of platelet–leukocyte aggregates and the release of more cytokines. These processes lead to endothelial dysfunction, heightened vascular permeability, and microvascular thrombosis, which are characteristic of sepsis-induced organ failure. Thrombocytopenia, which is linked to a higher risk of death, is often caused by too much platelet activation and consumption [13,14,15]. Because sepsis is dynamic and has many causes, early and accurate risk stratification is still very important for improving clinical outcomes. Patients may deteriorate rapidly within the first 24 h of ICU admission, highlighting the necessity for straightforward prognostic tools that enhance existing scoring systems and utilise routinely measured laboratory parameters. Even though blood-cell-derived ratios are not yet part of standard ICU severity scores, people are becoming more and more interested in how well they can predict things. In this context, the current study aimed to assess various platelet-derived indices as early indicators of in-hospital mortality in patients with sepsis or septic shock. We aimed to ascertain whether any of these markers could improve early risk assessment and facilitate prompt clinical decision-making in a population characterised by persistently elevated mortality rates through the simultaneous comparison of multiple ratios.

## 2. Materials and Methods

The research followed a prospective observational cohort design at a tertiary hospital ICU throughout 2023. The study included all adult ICU patients who received sepsis or septic shock diagnoses according to Sepsis-3 criteria which required suspected infection with acute organ dysfunction. The research team enrolled patients who met sepsis criteria through consecutive enrolment without implementing any specific study interventions while physicians made all treatment choices independently. We documented all essential clinical information and laboratory test results when patients first entered the ICU including complete blood count results with differential counts and serum biochemistry values.

The clinical laboratory at Spitalul Clinic Județean de Urgență Craiova (SCJU Craiova) conducted all blood tests using their state-of-the-art automated testing equipment which serves the entire hospital for standard clinical assessments in hematology and biochemistry and immunology and their associated fields. The laboratory operates through its trained staff who conduct standardized quality control tests to provide immediate results for patients who require urgent and hospital-based medical care. The researchers computed four platelet-based indicators from the collected data which included (1) C-reactive protein to platelet ratio (CPR) calculated by dividing serum C-reactive protein levels (mg/L) by platelet counts (10^9^/L) and (2) PLR obtained by dividing platelet counts by absolute lymphocyte numbers and (3) Platelet-to-white blood cell ratio (PWR) calculated by dividing platelet counts by total white blood cell counts and (4) Platelet-to-creatinine ratio (PCR) determined by dividing platelet counts by serum creatinine levels. The researchers selected specific ratio structures which represented different disease mechanisms. The CPR formula demonstrates how inflammation decreases platelet availability through its numerical structure which uses CRP as the numerator but PCR uses creatinine in its denominator to demonstrate platelet count relationship with kidney disease severity. Although the acronyms share similar letters, each ratio reflects a different biological interaction. The main research objective focused on tracking patient survival rates throughout their hospital stay. The primary outcome was all-cause in-hospital mortality. Patients were followed from the time of ICU admission until the date of hospital discharge or death, whichever occurred first. The researchers tracked all patients until they left the hospital or passed away. The study obtained approval from the institutional ethics committee because it used existing clinical data collected during standard patient care thus obtaining an informed consent waiver. The study did not require any interventions because it followed standard sepsis treatment protocols.

Descriptive statistics summarized baseline characteristics for total patients, but also for sepsis vs. septic shock groups. Continuous variables were presented as mean ± standard deviation (SD) for normal distribution or median [interquartile range, IQR] for non-normal distribution. Categorical variables were presented as frequencies. Kolmogorov–Smirnov test was used to assess normality. We used Student’s *t*-test (normal data) or Mann–Whitney U test (non-normal) for continuous variables, and Chi-square/Fisher’s exact test for categorical variables. To evaluate the four ratios’ potential as an early diagnostic marker for distinguishing mortality, univariate and multivariate logistic regression models were constructed. Variables demonstrating statistical significance (*p* < 0.05) in univariate analyses were included in multivariate binary logistic regression. Multicollinearity was assessed using Variance Inflation Factors (VIF). Due to the mathematical coupling of the indices (all sharing platelet counts or inflammatory markers), high VIF values (>10) were observed when including all ratios simultaneously. Consequently, we adopted a stepwise approach, constructing separate simplified multivariate models (Model B and Model C) to test the independent prognostic value of CPR against PLR and PWR individually, thereby mitigating overfitting and collinearity issues. ROC curves were generated to evaluate ratios’ diagnostic performance in identifying mortality, with the area under the ROC curve (AUC). The optimal cutoff point for the four ratios was determined based on Youden’s index (sensitivity + specificity − 1). Statistical analyses were conducted with R. We assessed AIC (Akaike Information Criterion) as key when comparing statistical models like logistic regression. All the statistical analyses were performed using the R statistical language, version 4.2.1 (www.r-project.org). Statistical significance was defined as a two-tailed *p* value of <0.05 [16].

To verify the adequacy of the sample size, a post hoc power analysis was performed using G*Power software (version 3.1.9.7).

## 3. Results

### 3.1. Study Population Characteristics

A total of 114 patients were enrolled, comprising 75 survival group and 39 non-survival group. The mean age of the study population was 71.25 ± 8.4 years, with no significant difference between survivors and non-survivors (*p* = 0.647). Males constituted 60.5% of the cohort, with no significant differences in sex distribution by mortality (*p* = 0.096).

Table 1 summarizes the laboratory characteristics. Non-survivors exhibited significantly higher CRP levels (140.0 ± 10.9 vs. 114.0 ± 21.4 mg/L; *p* < 0.0001) and CPR (1.59 ± 1.03 vs. 1.03 ± 0.92; *p* < 0.0001) than survivors. Platelet count was significantly lower in non-survivors (116.9 ± 53.0 vs. 146.6 ± 53.1 × 10^9^/L; *p* = 0.009), while lymphocyte count was also reduced (1.23 ± 0.5 vs. 1.57 ± 0.53 × 10^6^/L; *p* = 0.001).

The PWR was significantly lower in non-survivors (9.7 ± 8.3 vs. 11.77 ± 10.9, *p* = 0.047). PCR showed a trend toward lower values in non-survivors (82.7 ± 58.3 vs. 109.1 ± 71.4; *p* = 0.068) but did not reach significance.

The analysis was based on the differences observed in the primary biomarker, CPR, between non-survivors (1.59 ± 1.03) and survivors (1.03 ± 0.92). This yielded a medium-to-large effect size (Cohen’s d) of 0.61. Calculations indicated that a total sample size of 120 patients would be required to achieve a statistical power (1-β) of 0.95 at a significance level of 0.05. Consequently, our study population of 114 patients provides adequate statistical power (>90%) to detect meaningful differences in the primary prognostic indices.

### 3.2. ROC Analysis for Mortality

**Figure 1:**
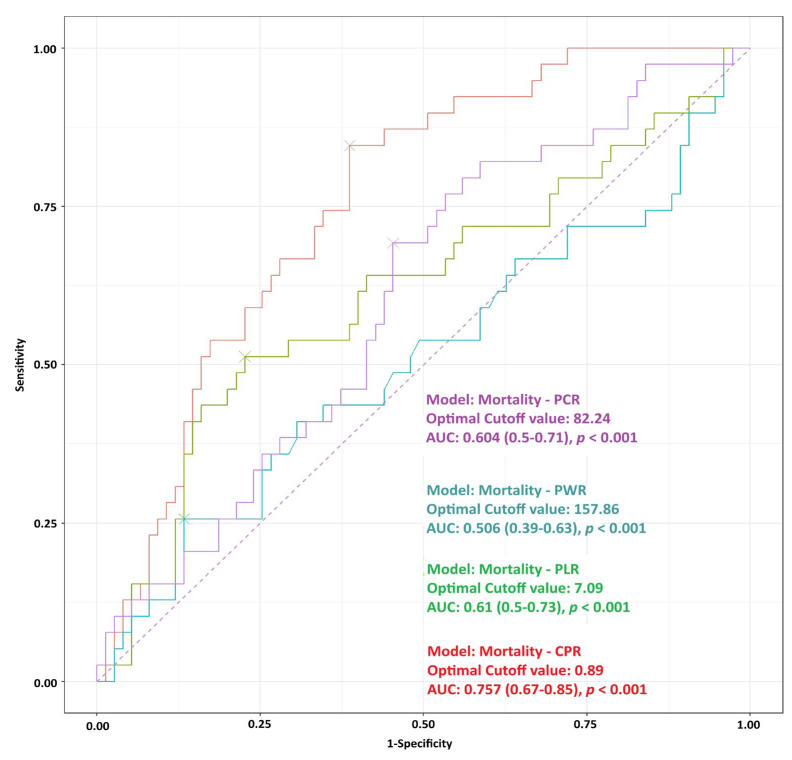
Receiver Operating Characteristic (ROC) analysis for mortality, evaluating the predictive performance of platelet-based biomarker ratios for in-hospital mortality.

Diagnostic performance of biomarkers ratios was performed using Receiver Operating Characteristic (ROC) analysis for mortality, evaluating the predictive performance of platelet-based biomarker ratios for in-hospital mortality (Figure 1).

Among the analyzed ratios, the CPR demonstrated the highest diagnostic accuracy, with an area under the curve (AUC) of 0.757 (95% CI: 0.669–0.846, *p* < 0.001). The optimal cutoff value for CPR was 0.886, above which patients were more likely to experience in-hospital mortality.

PLR and platelet-creatinine ratio (PCR) showed lower but statistically significant discriminatory ability. The AUC for PLR was 0.614 (95% CI: 0.498–0.729, *p* < 0.001), with an optimal cutoff of 7.10. Similarly, PCR yielded an AUC of 0.604 (95% CI: 0.497–0.712, *p* < 0.001) with an optimal cutoff of 82.24.

In contrast, PWR failed to demonstrate meaningful discrimination, with an AUC of 0.506 (95% CI: 0.388–0.625, *p* < 0.001) and a cutoff value of 157.86, indicating performance close to random chance.

### 3.3. Multivariate Logistic Regression for Mortality

To identify independent predictors of in-hospital mortality, a multivariate logistic regression model was constructed including the 4 platelet-based biomarker ratios. Among these, PLR and PWR were found to be statistically significant independent predictors of mortality, as shown in Table 2 (*p* < 0.001). PLR was positively associated with increased mortality risk (OR = 1.044; *p* < 0.001), indicating that for each unit increase in PLR, the odds of death increased by approximately 4.4%. PWR was negatively associated with mortality (OR = 0.570; *p* = 0.001), suggesting that higher PWR values conferred a protective effect. However, multicollinearity diagnostics revealed elevated variance inflation factors for PLR (VIF = 15.98) and PWR (VIF = 14.69), suggesting suppression effects and unstable estimates. While CPR showed strong discrimination in ROC analysis (AUC = 0.757), it did not remain significant in the adjusted model (*p* = 0.470), likely due to overlapping information with PLR and PWR.

Simplified or alternative models may be warranted to isolate the independent effect of each ratio. So, we rerun the logistic regression for CPR + PWR (Model B) and CPR + PLR (Model C) and report the best model. CPR remained statistically significant (*p* < 0.05) in both models, regardless of whether PLR or PWR was included. PLR and PWR were non-significant (*p* > 0.1) in both cases, suggesting no added independent predictive value when CPR is already in the model. VIF values were well below the threshold for concern (<2), so collinearity is not affecting the results in Model B and Model C.

The model including CPR and PLR yielded the best fit (AIC = 143), with CPR showing a significant association (OR = 1.98, 95% CI: 1.22–3.21, *p* = 0.006), while PLR was not significant (*p* = 0.199). Similarly, in the CPR + PWR model, CPR remained significant (*p* = 0.018), and PWR was non-significant (*p* = 0.962). No multicollinearity was detected (VIFs < 1.2). These findings support CPR as the most robust and reliable biomarker ratio for mortality prediction in this cohort.

### 3.4. ROC Curve for the Final Model

**Figure 2:**
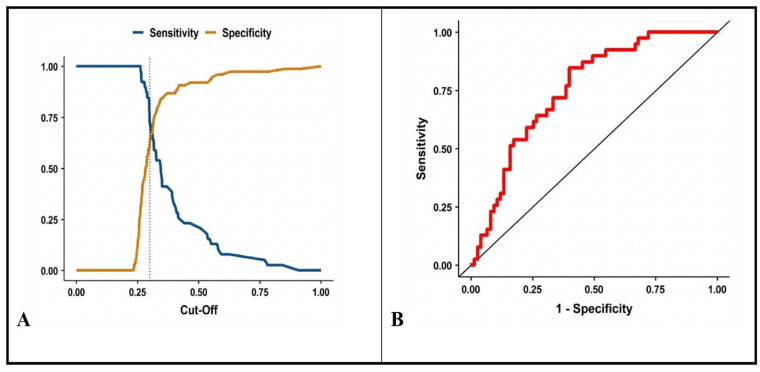
Diagnostic performance of the combined multivariate model (CPR + PLR) for predicting in-hospital mortality. (**A**). Plot of sensitivity and specificity against predicted probability cutoffs to determine the optimal threshold. (**B**). Receiver Operating Characteristic (ROC) curve for the combined model.

We calculated the combined AUC for the multivariate model using CPR and PLR with predicted probabilities as in Figure 2A,B. The predictive performance of the combined CPR + NLR logistic regression model for septic shock was reassessed using the optimal cutoff of 0.29, derived from the Youden Index. The combined model yielded an AUC of 0.772, indicating acceptable discriminatory ability (sensitivity = 69.2%, specificity = 73.3%). The model’s AUC of 0.772 demonstrates good overall discrimination, better than chance and better than the individual performance of CPR (AUC = 0.757) and PLR (AUC = 0.604) alone. The combination of these two markers enhances the model’s predictive power.

## 4. Discussion

Thrombocytopenia is a frequent complication in sepsis and serves as a key indicator of disease severity. A decline in platelet count within the first 72 h of ICU admission often signals the progression towards sepsis-induced coagulopathy or early disseminated intravascular coagulation (DIC). This relationship is incorporated into the SOFA score, which utilizes platelet count as a marker of coagulation failure. Extensive research has established that low platelet levels are linked to bad outcomes in sepsis [17,18,19]. In particular, platelet counts below roughly 100 × 10^9^/L serve as an independent predictor of heightened mortality [20,21]. Wang et al. further illustrated a curvilinear relationship wherein 28-day mortality significantly increased once platelet counts fell below this threshold [20]. Furthermore, dynamic platelet trends seem to provide more information than single platelet cutoffs [22]. Schupp et al. noted that a reduction exceeding 25% within the initial 5 days of treatment correlated with markedly elevated 30-day mortality [21]. Recent evidence indicates that the recovery of platelet counts following severe thrombocytopenia does not inherently correlate with enhanced survival [21,23,24].

These results are in line with what we found: non-survivors had much lower platelet counts when they were admitted than survivors (mean 117 vs. 147 × 10^9^/L). Platelet depletion resulting from consumption, destruction, and compromised production in sepsis indicates underlying microvascular thrombosis and immune-mediated clearance, mechanisms that lead to progressive organ failure and sustained thrombocytopenia [25,26].

In this context, our study assessed composite indices that amalgamate platelet counts with inflammatory or organ dysfunction indicators. The CPR was the most accurate way to predict death in the hospital. In our ROC analysis, a CPR cutoff of approximately 0.89 provided the best balance of sensitivity and specificity. Clinically, this threshold serves as an early warning system. A value exceeding 0.89 indicates a patient in whom the inflammatory stimulus (high CRP) is disproportionately high relative to the thrombotic reserve (lower platelets). This ‘mismatch’ identifies a high-risk phenotype that may require more aggressive monitoring for organ failure, distinct from patients with isolated thrombocytopenia or isolated CRP elevation. CRP, an acute-phase reactant, increases significantly during sepsis and has been linked to negative outcomes when elevated at admission [27,28]. In conjunction with platelet count, a recognised prognostic indicator, CPR signifies the simultaneous presence of substantial inflammation (elevated CRP) and haematologic decline (reduced platelets). This dual representation of inflammatory and coagulopathic burden elucidates its robust predictive capability in our study (AUC = 0.76), aligning with previous findings that demonstrated similar superiority over individual markers [29,30,31].

Although adult data on CPR in sepsis are scarce, analogous methodologies have been investigated in other patient cohorts. In neonates, an elevated CPR has been identified as an independent diagnostic and prognostic indicator of sepsis severity, indicating its applicability beyond neonatal care [32,33]. The behaviour of CPR is similar to that of other composite biomarkers, like the CRP-to-albumin ratio (CAR), which has shown to be useful for predicting outcomes in critically ill patients by measuring both inflammation and nutritional status [34]. Similarly, patients with pyogenic liver abscess and elevated CPR upon admission exhibited a heightened risk of developing sepsis and experiencing adverse clinical outcomes [35]. These studies collectively underscore CPR’s potential as a biomarker that reflects various physiopathological aspects concurrently.

In our cohort, every incremental increase in CPR correlated with a twofold increase in mortality risk, as determined by two-factor models. Moreover, CPR retained its significance even when evaluated in conjunction with PLR or PWR, indicating that it encompasses a substantial portion of the prognostic information provided by these alternative indices. A significant practical benefit of CPR is its dependence on standard laboratory tests, rendering it an economical and easily implementable bedside instrument for early risk stratification.

While the PLR has been investigated as a prognostic indicator in critical illness, its utility in sepsis appears. Early sepsis is frequently marked by lymphopenia due to apoptosis and immune dysregulation. Both very low and very high lymphocyte counts have been linked to higher death rates in critically ill patients, but lymphopenia is more common in sepsis. PLR may indicate thrombocytosis in relation to lymphocytes or, more frequently, lymphocytopenia; however, its behaviour can be affected by concurrent reductions in both cell types [36,37,38]. Shen et al. indicated that septic patients exhibiting PLR values exceeding 200 experienced elevated hospital mortality (OR ~1.3 for the highest quartile) [39]. In our study, however, PLR did not show a significant difference between survivors and non-survivors (median ~91 vs. 97), probably because both platelet and lymphocyte counts went down at the same time. PLR may also lose its ability to tell the difference in septic shock, where lymphopenia is usually very severe and lasts for a long time [40]. In multivariable models encompassing all indices, PLR initially seemed to be independently correlated with mortality; however, this association was nullified upon adjustment for CPR, suggesting multicollinearity and information overlap. Consequently, although PLR may exhibit a modest correlation with mortality, its additional predictive significance wanes in the context of a more robust composite marker like CPR. PLR may still be useful when CRP is not available or in multicomponent immunologic risk models. Changes in PLR during hospitalisation may also have prognostic value.

Similarly, PWR has recently garnered attention as a composite indicator of inflammatory equilibrium, predicated on the observation that resolution of inflammation is generally associated with declining white blood cell counts and rising platelet counts. Foy et al. suggested PWR as a universal prognostic indicator for various acute inflammatory conditions, such as COVID-19, acute heart failure, myocardial infarction, and stroke, demonstrating that diminished PWR values are associated with heightened mortality [41]. In theory, PWR could help tell the difference between inflammation that is getting better and inflammation that is getting worse. In our cohort, non-survivors exhibited lower PWR values in univariate analysis (7.1 vs. 9.5), consistent with previous research indicating that diminished platelet/WBC ratios forecast mortality [21]. Nonetheless, PWR did not differentiate mortality in ROC analysis (AUC ~0.50), suggesting restricted clinical applicability at a singular time point. In multivariate models, the perceived protective effect of elevated PWR (OR ~0.57) vanished upon the inclusion of CPR, indicating potential information overlap. The association between CRP (included in CPR) and WBC count may elucidate PWR’s reduced significance. Furthermore, PWR may possess enhanced prognostic significance when evaluated longitudinally, especially within the initial 48 h, a methodology corroborated by prior studies [21]. In summary, while low PWR corresponds with the pathophysiological premise that diminished platelet response during leukocytosis indicates adverse outcomes [42], its standalone impact on mortality prediction in sepsis seems restricted, necessitating additional research. In our study, the discrepancy between the univariate significance of PLR and PWR and their lack of significance in multivariate models is likely due to collinearity. Since CPR, PLR, and PWR all share components (platelets), they capture overlapping biological information. Our multivariate analysis suggests that CPR is the mathematically dominant variable, absorbing the predictive variance that PLR and PWR offer in isolation. This implies that CPR is a more comprehensive marker of the sepsis-induced immuno-thrombotic failure than the other ratios.

We also looked at PCR, which is meant to show both kidney problems and blood clotting problems at the same time. Both of these are important parts of how sepsis works. Acute kidney injury is prevalent among septic patients, and increased creatinine levels correlate with elevated severity scores. A decreased PCR may signify the simultaneous presence of renal impairment and thrombocytopenia, indicating multi-organ failure [43,44]. In our study, PCR levels were lower in non-survivors, although this difference did not achieve statistical significance (*p* ≈ 0.07). The results align with the SOFA framework, which indicates that renal and coagulation dysfunction collectively increase mortality risk. The current literature regarding PCR in sepsis is limited; however, studies involving cirrhotic patients have suggested analogous ratios as indicators of unfavourable outcomes [45,46]. Zhao et al. also found that platelet-related measures, like PCR and mean platelet volume, could predict 28-day mortality in septic patients on their own [30]. It is important to note that different studies define PCR in different ways, but all of them combine platelet count with signs of organ dysfunction [45]. Our findings indicate that PCR may possess prognostic potential; however, more extensive studies are required to confirm its clinical relevance and establish standardised definitions and thresholds [47,48].

The strengths of our study encompass its prospective design, the inclusion of all consecutive sepsis and septic shock patients, and its reliance on universally accessible laboratory tests. The direct comparison of various platelet-based indices introduces innovation to the literature and substantiates CPR as a superior prognostic indicator upon ICU admission. In a clinical setting, CPR may help find high-risk patients early on. For instance, our research shows that a patient with a CRP level of 200 mg/L and a platelet count of 100 × 10^9^/L would have a CPR of 2.0, which means they are at a much higher risk of getting worse. Such information may facilitate the intensification of monitoring and treatment. Also, keeping an eye on platelet-based indices over time may help doctors figure out if a patient is getting better or if their apparent stability is hiding a decline in their health.

The study population did not have access to standard clinical testing for the established inflammatory biomarkers which included procalcitonin and interleukin-6. The medical field depends on procalcitonin to determine infection severity and track antibiotic treatment and IL-6 functions as the first cytokine marker for sepsis patient diagnosis. The two biomarkers need complex testing methods, which make them expensive to obtain for general medical use. The risk evaluation process through CPR depends on standard clinical data which provides healthcare providers with a simple method to start assessing patients. Future research needs to establish a direct comparison between CPR and procalcitonin and IL-6 to determine their individual prognostic value.

### Limitations

This study has several limitations that should be acknowledged. The interpretation of our results must be viewed within the context of the study’s constraints. First, it was done at only one centre with a small number of participants and a small number of deaths, which makes the analyses less statistically powerful and may make them less applicable to other situations. The derived cutoff values may be specific to our local population and practice patterns, requiring validation in larger, multicenter cohorts before clinical adoption. The low events-per-variable ratio also makes it more likely that multivariable models will overfit, even though we tried to fix collinearity by using simpler model structures.

Second, the biomarker assessment was only based on admission values. Because sepsis is a changing condition, taking multiple measurements over time, especially of CPR, PLR, and PWR, may give better prognostic information than taking measurements at just one point in time. Future studies should look at how well temporal trends can predict outcomes, including changes that happen in the first 24 to 48 h after a patient is admitted to the ICU. We relied on a single measurement of biomarkers at ICU admission. Sepsis is a highly dynamic syndrome; a ‘snapshot’ view fails to capture the trajectory of the disease. It is possible that the ‘delta’ (change) in CPR over the first 48 h is a superior predictor than the admission value alone.

Third, the study did not compare platelet-based indices to other well-known biomarkers or severity scores, like lactate or procalcitonin. To avoid overfitting, we restricted the number of covariates, which meant we could not adjust for potential confounders such as the SOFA score, APACHE II, or specific comorbidities (e.g., malignancy, chronic kidney disease). Adding CPR to existing prognostic frameworks could give us more information, but that was not the focus of this study.

Fourth, even though we tried to reduce bias by enrolling patients one after the other, we cannot rule out unmeasured confounders. Changes in the timing of antibiotics, resuscitation methods, or other clinical interventions may have had an effect on outcomes and biomarker behaviour.

Lastly, the observational design makes it impossible to draw causal conclusions. The links found here are correlations, not mechanistic relationships. More multicenter studies with a wider range of people are needed to confirm that platelet-derived ratios can be used to predict outcomes.

## 5. Conclusions

In this study of critically ill patients with sepsis, CPR was found to be the best platelet-derived index for predicting early death. CPR showed consistent and independent prognostic values, which were due to the combined effects of systemic inflammation and sepsis-related low platelet counts. CPR is a useful and cost-effective tool that could easily be used as part of early risk assessment when a patient is admitted to the ICU because it is based on routine lab tests.

PLR and PWR, on the other hand, did not do a good job of telling the difference when compared to CPR. This suggests that these markers capture a lot of the same biological information. PCR may be linked to multiorgan dysfunction, but it was not statistically significant, so more research is needed with larger groups.

In general, our results show that CPR could be a simple additional biomarker to help with early prognostic stratification in sepsis. Future studies should check these results in populations from more than one centre, look into taking multiple CPR measurements over time, and see how useful it is to add CPR to established prognostic models like SOFA or APACHE II. These kinds of efforts may help improve risk stratification and lead to timely, personalised care for this condition, which has a high death rate.

## Figures and Tables

**Table 1:** Baseline demographic and laboratory characteristics of the study population stratified by mortality.

Characteristics	Total (n = 114)	Mortality		
		Survivors (n = 75)	Non-Survivors (n = 39)	*p*-Value
Age, years	71.25 ± 8.4 71 (65.8–77)	71.11 ± 8.2 72 (65–76)	71.5 ± 9 71 (67–79)	0.647
Sex, male	69 (60.5%)	49 (65.3%)	20 (51.3%)	0.096
Shock status				
Septic shock	38 (33.3%)	17 (22.7%)	21 (53.8%)	0.001
Sepsis	76 (66.7%)	58 (77.3%)	18 (46.2%)	
**Biochemical parameters**				
CRP (mg/L)	122.9 ± 22.2 128.9 (106.1–137.8)	114.04 ± 21.4 120.5 (94.1–130.5)	140 ± 10.9 138.5 (131.5–148.7)	<0.0001
Lymphocytes (10^6^ cells/L)	1.5 ± 0.5 1.4 (1.2–1.8)	1.57 ± 0.53 1.6 (1.3–1.9)	1.23 ± 0.5 1.2 (1–1.6)	0.001
WBC (10^6^ cells/L)	15.4 ± 4.7 16.3 (13.4–18.5)	15.44 ± 4.34 16.3 (13.4–18.2)	15.4 ± 5.31 16.4 (13.1–19)	0.558
PLT (10^9^ cells/L)	136.4 ± 54.7 140 (100–169)	146.6 ± 53.1 146 (118–189)	116.9 ± 53.04 118 (73–158)	0.009
Neutrophils (10^6^ cells/L)	12.2 ± 4.1 13.9 (11.3–16.1)	13.2 ± 3.84 13.8 (11.3–16)	13.3 ± 4.7 14.2 (10.5–17.1)	0.525
Creatinine (µmol/L)	168.0 ± 123.8 114.9 (85.7–207.7)	168.0 ± 123.8 106.1 (84.9–247.5)	168.0 ± 114.9 159.1 (85.7–185.6)	0.774
**Derived platelet-based indices (calculated ratios)**				
CPR	1.22 ± 0.99 0.9 (0.6–1.3)	1.03 ± 0.92 0.76 (0.6–1.1)	1.59 ± 1.03 1.24 (0.9–1.77)	<0.0001
PLR	123.2 ± 119.8 93.3 (62.5–138.8)	120.4 ± 117.7 91 (66.7–138.3)	128.7 ± 125.3 96.9 (54.3–157.9)	0.912
PWR	11.1 ± 10.1 9.2 (6–12.2)	11.77 ± 10.9 9.5 (7.4–12.6)	9.7 ± 8.3 7.1 (4.8–11.8)	0.047
PCR	100 ± 68.1 76.6 (51.8–137.1)	109.1 ± 71.4 93 (55.3–147.5)	82.7 ± 58.3 68.4 (45.1–104.9)	0.068

CRP—C-reactive protein, WBC—White blood cells, PLT—Platelet; CPR—CRP/platelet ratio; PLR—platelet-lymphocyte ratio; PWR—platelet-WBC ratio; PCR—platelet-creatinine ratio. Creatinine values were converted from conventional units (mg/dL) to SI units (µmol/L) using the conversion factor 1 mg/dL = 88.4 µmol/L. Continuous variables are presented as mean ± SD (standard deviation) and median (IQR, interquartile range).

**Table 2:** Summary of final models.

Model	Variable	Multivariate		AIC	R^2^_McF_	AUC
		OR (95%CI)	*p*-Value			
Model A	CPR PWR PLR PCR	0.915 (0.70–2.14) 0.570 (0.42–0.78) 1.044 (1.02–1.07) 0.997 (0.99–1.01)	0.47 <0.001 <0.001 0.503	125	0.214	0.826
Model B	CPR PWR	1.798 (1.11–2.92) 0.999 (0.96–1.04)	0.018 0.962	144	0.055	0.754
Model C	CPR PLR	1.977 (1.22–3.21) 1.002 (0.99–1.01)	0.006 0.199	143	0.066	0.772

AIC—Akaike Information Criterion; R^2^_McF_—McFadden’s R^2^.

## Data Availability

The data from this study is not available to the public because of ethical and privacy regulations. The corresponding author can provide access to anonymized datasets after receiving a reasonable request and obtaining approval from our institutional ethics committee.

## Abbreviations

The following abbreviations are used in this manuscript:

**Abbreviation**	**Full Term**
ICU	Intensive Care Unit
CRP	C-reactive protein
CPR	C-reactive protein-to-platelet ratio
PLR	Platelet-to-lymphocyte ratio
PWR	Platelet-to-white blood cell ratio
PCR	Platelet-to-creatinine ratio
WBC	White blood cell count
SOFA	Sequential Organ Failure Assessment
qSOFA	Quick Sequential Organ Failure Assessment
NLR	Neutrophil-to-lymphocyte ratio
RDW	Red cell distribution width
MPV	Mean platelet volume
DIC	Disseminated intravascular coagulation
AUC	Area under the receiver operating characteristic curve
ROC	Receiver Operating Characteristic
SD	Standard deviation
IQR	Interquartile range
AIC	Akaike Information Criterion
OR	Odds ratio
CI	Confidence interval
